# Systematic Review and Meta-Analysis of Wound Bundles in Emergency Midline Laparotomy Identifies That It Is Time for Improvement

**DOI:** 10.3390/life11020138

**Published:** 2021-02-11

**Authors:** Gearóid Mc Geehan, Itoro M. Edelduok, Magda Bucholc, Angus Watson, Zsolt Bodnar, Alison Johnston, Michael Sugrue

**Affiliations:** 1 Donegal Clinical Research Academy, Letterkenny University Hospital, F92AE81 County Donegal, Ireland; alison.johnston@hse.ie (A.J.); Michael.sugrue@hse.ie (M.S.); 2 School of Medicine, University of Limerick, V94T9PX Limerick, Ireland; 3 Department of Surgery, Letterkenny University Hospital, F92AE81 County Donegal, Ireland; itoro.edelduok@ucdconnect.ie (I.M.E.); Zsolt.Bodnar@hse.ie (Z.B.); 4 Intelligent Systems Research Centre, School of Computing, Engineering and Intelligent Systems, Ulster University (Magee Campus), Northern Ireland BT48 7JL, UK; m.bucholc@ulster.ac.uk; 5 Raigmore Hospital, NHS-Highland, Inverness IV2 3DZ, UK; angus.watson@nhs.scot; 6 Emergency Surgery Outcome Advancement Project, Letterkenny University Hospital, F92AE81 County Donegal, Ireland

**Keywords:** emergency surgery, laparotomy, surgical site infection, midline incision, wound bundle

## Abstract

Background: Emergency midline laparotomy is the cornerstone of survival in patients with peritonitis. While bundling of care elements has been shown to optimize outcomes, this has focused on elective rather than emergency abdominal surgery. The aim of this study was to undertake a systematic review and meta-analysis of factors affecting the development of surgical site infection (SSI) in patients undergoing midline emergency laparotomy. Methods: An ethically approved, PROSPERO registered (ID: CRD42020193246) meta-analysis and systematic review, searching PubMed, Scopus, Web of Science and Cochrane Library electronic databases from January 2015 to June 2020 and adhering to PRISMA guidelines was undertaken. Search headings included “emergency surgery”, “laparotomy”, “surgical site infection”, “midline incision” and “wound bundle”. Suitable publications were graded using Methodological Index for Non-Randomised Studies (MINORS); papers scoring ≥16/24 were included for data analysis. The primary outcome in this study was SSI rates following the use of wound bundles. Secondary outcomes consisted of the effect of the individual interventions included in the bundles and the SSI rates for superficial and deep infections. Five studies focusing on closure techniques were grouped to assess their effect on SSI. Results: This study identified 1875 articles. A total of 58 were potentially suitable, and 11 were included after applying MINORS score. The final cohort included 2,856 patients from eight countries. Three papers came from the USA, two papers from Japan and the remainder from Denmark, England, Iran, Netherlands, Spain and Turkey. There was a 32% non-significant SSI reduction after the implementation of wound bundles (RR = 0.68; CI, 0.39–1.17; p = 0.16). In bundles used for technical closure the reduction in SSI of 15% was non-significant (RR = 0.85; CI, 0.57–1.26; p = 0.41). Analysis of an effective wound bundle was limited due to insufficient data. Conclusions: This study identified a significant deficit in the world literature relating to emergency laparotomy and wound outcome optimisation. Given the global burden of emergency general surgery urgent action is needed to assess bundle’s ability to potentially improve outcome after emergency laparotomy.

## 1. Introduction

Emergency laparotomy, while a potentially life-saving procedure in patients with overt sepsis or bleeding, is associated with significant mortality and morbidity [1,2]. Surgical site infection (SSI), both superficial and deep, occurs in up to 35% of patients undergoing emergency abdominal surgery [3]. Increasingly, performance improvement programmes are focusing on optimising laparotomy outcomes in order to reduce morbidity and mortality, particularly in relation to surgical site infection [4,5]. SSIs are not only a source of both inconvenience and added cost, but they may also result in adverse oncological outcomes [6,7].

A number of risk factors for SSIs have been clearly recognised and many studies identify interventions whose implementation reduces the relative risk of complications [8,9]. When combined, these interventions form a bundle. Wound bundles have been shown to exert significant improvements in outcomes in elective surgery [7,10,11]. Reducing SSIs requires a team approach, involving all providers, in every phase of care, with a cumulative additive benefit of each aspect in the bundle. A wound bundle, in general, will have more than three components and extend from pre-operative care through to rehabilitation. Fundamental to a bundle is timely antibiotic administration, glucose control, prevention of hypothermia, hypotension and hyperglycaemia combined with newer concepts including incisional negative pressure therapy [12] and wound protective devices [13,14].

Wound bundles exert both short and long-term impact on SSI and also have the potential to improve oncological outcomes in cancer patients [15]. It has been suggested that wound bundles should be documented and used in over 90% of emergency laparotomies [16]. While meta-analyses have been undertaken on the efficacy of wound bundles in elective surgery [17], none have been conducted on emergency abdominal surgery.

The primary aim of this study was to perform a meta-analysis of wound bundles that may reduce the development of SSI and the secondary aim was to perform a meta-analysis of elements in wound bundles that may reduce rates of SSI in patients undergoing emergency midline laparotomy.

Surgical emergencies pose a considerable health burden with over 3 million emergency admissions in the US and globally it has been estimated that 258,300 patients die during their emergency surgical care annually [18,19,20]. Patients requiring emergency general surgery (EGS) are often critically ill on presentation, often with multiple pre-existing comorbidities and over 35% of EGS are over 70 years of age. Emergency surgery carries high rates of morbidity and mortality [20]. Patients undergoing EGS procedures are up to eight times more likely to die than those undergoing the same procedure electively [21]. EGS admissions and costs are projected to increase 45% to $41.20 billion annually by 2060 using US Census projections [22]. Despite the increasing use of laparoscopy, it is laparotomy that is the defining procedure in the 30% of admitted EGS patients who require surgery. This mandates that the technical approach and the overall bundle approach to laparotomy undergo rigorous process and outcomes evaluation.

## 2. Methods

### 2.1. Search Strategy and Study Eligibility

A systematic review and meta-analysis of the literature was undertaken to incorporate articles relating to emergency midline laparotomies, surgical site infections and surgical site wound bundles. Existing research that optimises wound care in emergency midline laparotomies was reviewed to determine current bundle strategies.

A systematic review and meta-analysis of all published English articles was conducted using the PubMed version of Medline, Scopus, Web of Science and the Cochrane Library electronic databases. To assess contemporary evidence only studies published between 1 January 2015 and 16 June 2020 were included. A literature search was conducted using subject headings, keywords and free text terms for the keywords and their variations. MeSH terms were used to search Pubmed and Scopus. The reference sections of reviewed studies were examined for further papers not identified by the initial search strategy. Citations were collated with Microsoft excel and duplicates removed. While a laparotomy can be performed through many different incisions [23,24], the vast majority are through a midline incision which was the sole focus of our study.

### 2.2. Inclusion and Exclusion Criteria

The methods of the analysis and inclusion criteria were specified in advance to avoid selection bias and documented in a protocol which was registered and published with the PROSPERO database (International Prospective Register of Systematic Reviews, www.crd.york.ac.uk/prospero, registration number: CRD42020193246 on 27 July 2020). This meta-analysis adhered to the Preferred Reporting Items for Systematic Reviews and Meta-Analyses (PRISMA) statement [25].

The Centers for Disease Control and Prevention (CDC) definitions for surgical site infection were used, which classifies them as superficial, deep or organ/space [26].

The Institute for Healthcare Improvement states that a care bundle is a structured way of improving the processes of care and patient outcomes: a small, straightforward set of evidence-based practices (generally three to five) that, when performed collectively and reliably, have been proven to improve patient outcomes [27]. An SSI bundle must have a minimum of three elements.

To be included, studies had to satisfy the following pre-determined criteria: (1) include emergency midline laparotomy only; (2) report post-operative surgical site infections (as either primary or secondary endpoint) and wound bundles; (3) studies with pre-, intra- and post-intervention SSI data; (4) design was a randomised controlled trial, prospective observational or retrospective cohort study; (5) reporting ten or more patients; (6) full text articles in the English language.

Studies were excluded if they (1) were designed as case reports, letters, or with <10 patients; (2) considered only organ space SSI; (3) included patients with an open abdomen; (4) included laparotomy converted from laparoscopy; (5) did not compare results to pre-intervention SSI rates.

### 2.3. Eligibility Assessment and Data Extraction

We screened titles and abstracts, reviewed full texts and extracted data. Eligibility assessment was performed independently in a blinded standardised manner by two reviewers (GMG and IE). We resolved disagreements by consensus and if no agreement could be reached, a third reviewer (AJ) decided.

Two reviewers (GMG and IE) independently assessed each published study for the quality of study design and risk of bias by using standardised pre-piloted forms, methodological index for non-randomised studies (MINORS) score [28]. A MINORS score of ≥16 out of 24 for comparative and ≥10 for non-comparative was considered the standard for inclusion.

A standardized data sheet was developed. Information was extracted from each included study on SSI classifications, bundle elements, bundle adherence rates, study design, country, study length, cohort sizes, and SSI rates pre-, intra- and post-intervention.

The primary outcome in this study was SSI rates following the use of wound bundles. Secondary outcomes were the effect of individual interventions included in the bundles and the SSI rates for superficial and deep infections.

### 2.4. Statistical Analysis

For comparison of SSI rates pre-and post-intervention Risk Ratios (RR) were calculated using Review Manager Version 5.4 (Copenhagen: The Nordic Cochrane Centre, The Cochrane Collaboration, 2008). Meta-analyses were performed by computing the RR using fixed-effect models, depending on the heterogeneity of studies. A RR and Confidence interval (CI) of >1.0 indicated greater risk of an adverse event occurring in the experimental group.

Heterogeneity was assessed using the I^2^ statistic where a value greater than 50% was considered high and a random-effect model was then used to combine variables of interest [29]. RR and 95% Confidence Intervals (CI) were calculated for each classification of SSI, along with the p-value for which a value <0.05 represented statistical significance.

A leave-one-out sensitivity analysis was used to estimate individual study effect on meta-analysis results of the rest of the studies.

### 2.5. Assessment of Risk of Bias in Included Studies

**Figure 1:**
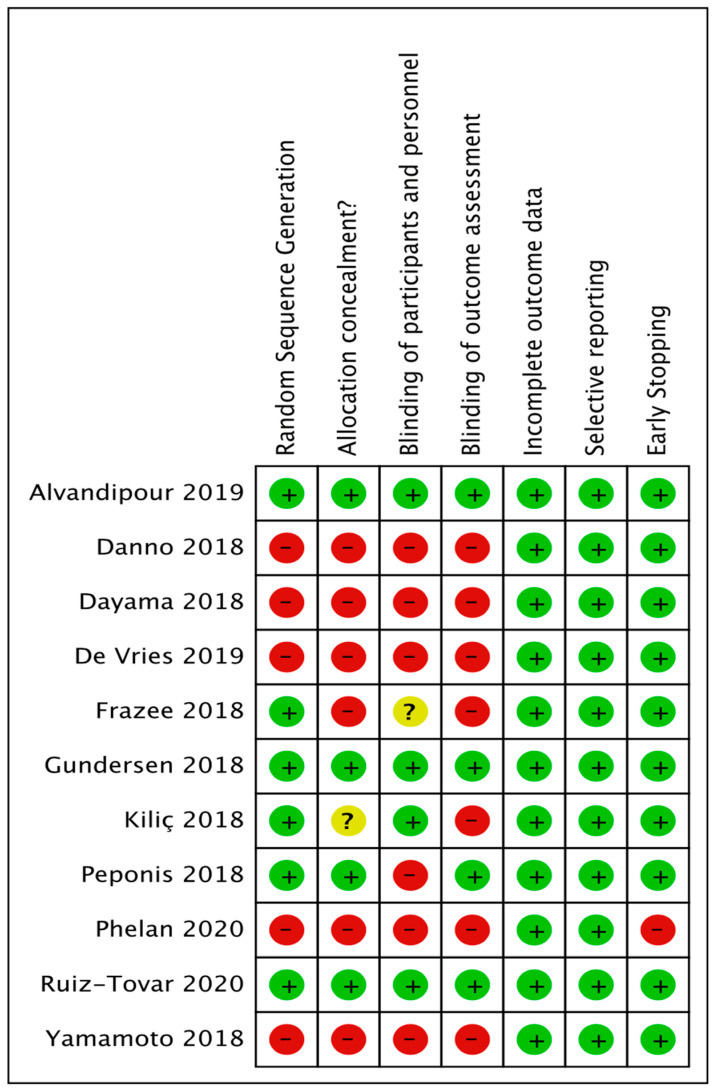
Risk of bias summary: review authors’ judgements about each risk of bias item for each included study.

Cochrane “Risk of bias” tool assessed bias as specified in chapter 8 of the Cochrane Hand-book for Systematic Reviews of Interventions [30], for the following domains:

(1) random sequence generation; (2) allocation concealment; (3) blinding of participants and personnel; (4) blinding of outcome assessment; (5) incomplete outcome data; (6) selective reporting bias; (7) and early stopping. As demonstrated in Figure 1.

## 3. Results

**Figure 2:**
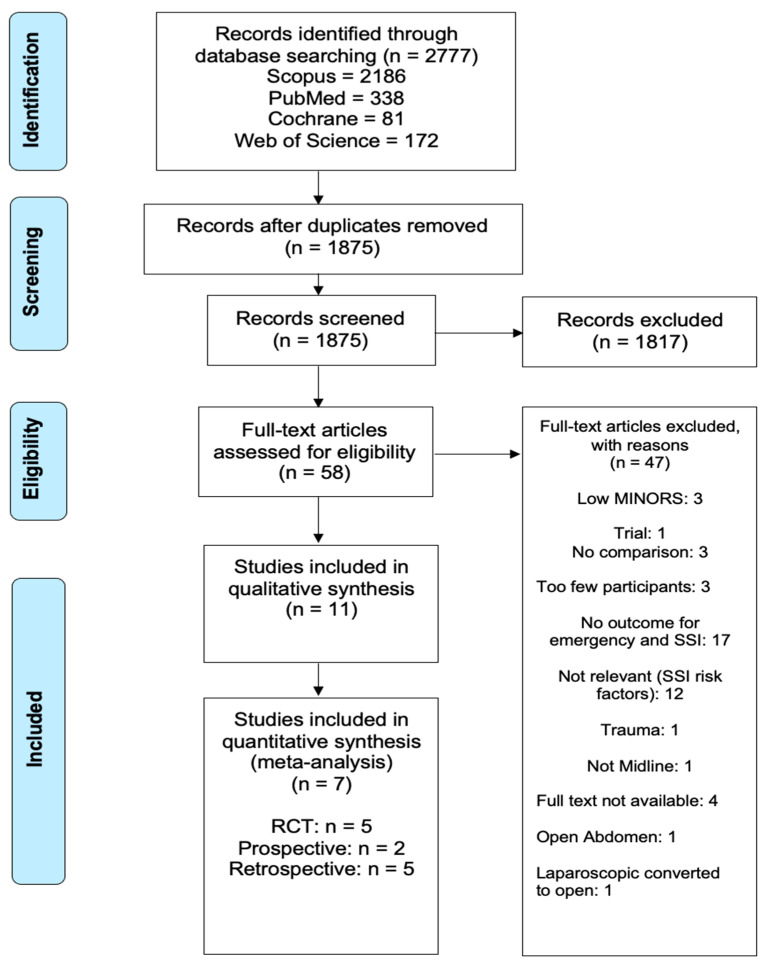
Identification, review and selection of articles included in the meta-analysis for impact of wound bundles on surgical site infections in emergency midline laparotomy surgery.

Out of the 1875 articles assessed as part of this systematic review and meta-analysis, a total of 11, spanning eight countries and three continents, were ultimately identified as potential candidates for systematic review. After excluding four of these 11 from the meta-analysis due to the absence of any overlapping wound-bundle elements, seven met criteria for meta-analysis (final cohort n = 2856) (Figure 2). Only two of these studies directly addressed surgical wound bundle implementation and the effects on SSI rates; the remaining five discussed various abdominal closure techniques effect on SSI rates. The characteristics of the studies included are shown in Table 1.

### 3.1. Outcomes

Of the seven included studies in the meta-analysis, only two contained data on the pre-implementation and post-implementation of a surgical wound bundle and its effect on overall SSI.

**Figure 3:**
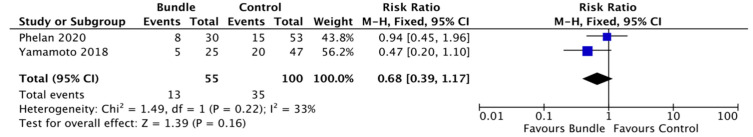
Forest plot: surgical wound bundle vs. control to reduce the risk of surgical site infections.

The meta-analysis, while showing a reduction in the risk of SSIs by 32% following the implementation of a wound bundle (23.6%, 13/55) versus no bundle (35%, 35/100) (Figure 3), was not statistically significant (RR = 0.68; CI, 0.39–1.17; p = 0.16).

The contents of the wound bundles varied and Phelan used the following elements to his wound bundle, divided into three phases [39]: pre-op (patients advised against hair removal, an on-table “social wash” and prophylatic antibiotics); intra-op (ensure normothermia, reduce movement theatre (door locks), Chloraprep scrub, antibacterial sutures and glove/gown/instrument/drape change for skin closure); post-op (wound care advice leaflet, best practise guidelines for SSI treatment and wound inspection stickers for assessment at discharge).

Yamamoto on the other hand focused solely on an intra-op wound bundle with the following elements [41]: Triclosan-coated polydioxanone antimicrobial sutures; irrigation: >500 mL of warm saline; wound dressing: cyanoacrylate tissue adhesive; drain: subcutaneous is not inserted; antibiotics: administered 30 min prior to surgery and continued every 3 h thereafter during surgery.

**Figure 4:**
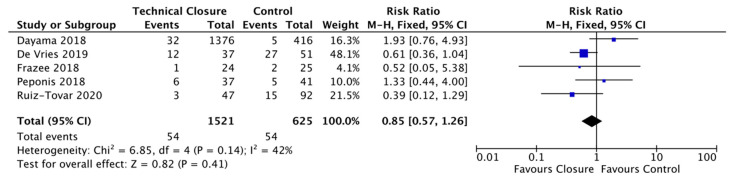
Forest Plot: Technical Closure Bundle Vs. Control to reduce the risk of Surgical Site infections.

We grouped the abdominal closure techniques in the remaining five studies into one technical closure bundle and examined their combined effect on SSI. Our technical closure group showed a non-significant reduction of 15% in SSI rates (3.55%, 54/1521) compared to the control group (8.64%, 54/625) (RR = 0.85; CI, 0.57–1.26; p = 0.41) (Figure 4). There was a moderate level of heterogeneity between trials (I^2^ = 42%) [33,34,35,38,40].

**Figure 5:**
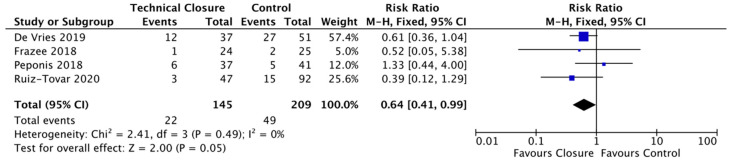
Forest plot: technical closure bundle Vs. control to reduce the risk of surgical site infections following leave one out sensitivity.

Following leave-one-out sensitivity analysis, it was shown after leaving out Dayama’s study that our technical closure bundle became significant and showing a reduction in SSI of 36% (RR = 0.64; CI, 0.41–0.99; p = 0.05) (Figure 5).

The meta-analysis of the five grouped abdominal wall closure studies are limited by their small number and heterogenicity [33,34,35,38,40]. Dayama examined complete skin closure versus skin-open [33]. There were, however, no participants for the skin-open arm of the study, due to there being no superficial SSIs in the US; this is in a bid by hospitals to avoid incurring a financial penalty, as highlighted by Ball [42].

Frazee examined incisional negative pressure wound therapy (iNPWT) on open and closed wounds; with the small numbers in this trial, it was found to be insignificant [35]. Ruiz-Tovar found that triclosan coated barbed suture was more effective against polydioxanone loop suture, but was found to be insignificant [40].

DeVries found that small bite technique was superior to continuous fascial closure, but the difference detected proved insignificant [34]. Peponis found continuous fascial closure compared to interrupted fascial closure had a non-significant reduction in SSI from 16.2% to 12.2% (RR = 1.33; CI, 0.44–4.00; p = 0.61) [38].

### 3.2. Overall SSI

A total of 11 studies reported a change in the rate of overall SSI after the implementation of a surgical wound bundle or a surgical bundle element. The total cohort size of all studies of the pre-implementation group was 1197, and the size of post-implementation group was 2046. This study reported a non-significant decrease in SSI rates after the implementation of a either surgical wound bundle or a surgical wound bundle element (i.e., 7.8%, 160/2046) versus control (15.9%, 190/1197) (RR = 0.88; CI, 0.72–1.08; p = 0.24).

### 3.3. Superficial SSI Rates

Superficial SSI rates were discussed in two studies, which had a total of 1892 patients. Kilic examined 100 patients and reported a 35% reduction in SSI (RR = 0.65; CI, 0.34–1.24; p = 0.19) [37]. Dayama’s cohort of 1792 patients reported superficial SSI; however, the skin wound was left open for the control arm of the study and a comparative difference in the rate of superficial SSIs could not be recorded [33].

### 3.4. Deep SSI Rates

Deep SSI rates were reported in four studies of 2050 patients, with matched pre- and post-intervention patients. This study has reported an overall significant decrease in deep SSI rates after the implementation of a surgical wound bundle or a surgical wound bundle element (2.9%, 42/1476) compared to the control (9.1%, 52/574) (RR = 0.6; CI, 0.38–0.95; p = 0.03).

### 3.5. Bundle Element Results

There were 12 factors that influenced the SSI rates as outlined in Table 2. In a study of 26 patients, Alvandipour identified an SSI rate of 29% in the control group (FiO_2_ 30%) and 16% in the comparative group (FiO_2_ 80%) [31]. Danno in a study of 47 patients, reported an SSI incidence of 10.7% when the NPWT technique was used for delayed primary closure, compared to the 63.2% incidence recorded for primary suturing (p < 0.001) [32]. Dayama in a study of 1792 participants, reported a deep SSI rate of 2.3% in the complete closure cohort and 1.2% in the incision skin open group (p = 0.15) [33]. In his 88-patient study of the small bites technique versus large bites fascial closure, de Vries found that 35% developed SSIs using the small bites technique compared to 57% with the large bites technique [34]. Frazee then examined 49 patients as part of an RCT and showed a 4.2% rate of SSI for incisional NPWT and skin open compared to 8% for incisional NPWT and skin closed [35]. Gundersen in a study of 382 participants, explored SSI using a fluid infusion index (FII) [36]. He identified an SSI rate of 18.9% for the lower tertile (<2.71 mL/kg/h) and 22.1% for the higher tertile (>5.64 mL/kg/h) in comparison with the middle tertile of 17% (2.71–5.64 mL/kg/h). Gundersen also examined the effects of intra-operative temperature on SSI. Hyperthermia (>37.6 °C) was shown to have an insignificant SSI rate of 22.7% (p = 0.34), hypothermia (<35.4 °C) had a significant SSI rate of 39.1% (p = 0.004) and normothermia (35.4 °C–37.6 °C) had an SSI rate of 17% [36]. Kilic investigated the effects of hypothermic compression on SSIs in 100 patients, finding a reduction 22%, in comparison to conventional sterile compression 34% [37]. Peponis examined the correlation between closure techniques and the rate of SSI, comparing interrupted fascial closure 16.2% versus continuous fascial closure 12.2% [38]. Ruiz-Tovar then studied the effects of closure sutures on deep SSI rates, comparing triclosan-coated barbed suture 6.4% with the control of polydioxanone loop suture 16.3% [40]. Phelan and his cohort of 83 patients, examined the rate of SSI with bundles 26.7% and without bundles 28.3% [39]. Lastly, Yamamoto, similarly to Phelan, examined the effects of bundles on SSIs, identifying an SSI rate of 20% with a bundle and 42.6% without [41].

### 3.6. Patient Demographics and Surgery Indication

Four of the studies’ information on patient demographics and indications for surgery were unobtainable as elective and emergency cohorts were not sub categorized [31,34,36,39].

Danno’s population (n = 47) had a median age of 68; 22 were males and all indications were lower gastrointestinal (GI) perforation [32]. In Dayama’s cohort of emergency colectomies (n = 1792), 870 were male, the median age was 63 [33]. Frazee’s (n = 49) patients’ characteristics had a median age of 57, 31 males and the indications that were given for surgery were gastroduodenal, small bowel and colonic perforations [35]. Kiliç, in a study of 100 patients, consisted of 41 males whose median age was 53 and surgical indications were GI perforation, intestinal obstruction, acute cholecystitis-cholangitis, incarcerated ventral hernia, acute appendicitis, liver/spleen laceration, strangulated inguinal hernia, GI haemorrhage, acute necrotizing pancreatitis and mesenteric ischemia [37]. In Peponis (n = 78) it is unable to determine gender and age numbers and the primary indications found for surgery were small bowel obstruction, colonic perforation and *C. difficile* colitis [38]. Ruiz-Tovar’s group of 139 had a similar median age cohort of 65, 79 of which were male patients, and patients underwent surgery for bowel obstruction, perforated diverticulitis, perforated neoplasm and acute bowel ischemia [40]. Lastly, in Yamamoto’s collection (n = 72), with a median age of 72, 37 males partook in the trial and all of the operations performed were for colorectal perforation [41].

## 4. Discussion

Emergency surgery accounts for 10% of hospital admissions and has one of the highest mortalities in medicine [21]. More than 30,000 patients undergo an emergency laparotomy each year in NHS hospitals in England and Wales [4]. More than 3 million patients are admitted to US hospitals annually for EGS for diseases such as perforated viscus, appendicitis and cholecystitis [43]. As part of management of emergency surgery, a laparotomy with its inherent sepsis and haemorrhage control is the mainstay of treatment. Getting this right is essential as it is associated with significant complications. Mortality in EGS patients is 13% compared to 3% for elective surgery, with major complications in 33% of EGS patients compared to 13% in elective surgery. It is estimated that the cost of EGS care in the US alone will reach USD 41 billion by 2060 [44].

Wound bundles did not demonstrate a significant reduction in SSI for emergency laparotomy in our study, mostly due to the small numbers of published wound bundle evaluations in patients undergoing emergency laparotomy.

EGS patients pose a specifically high-risk challenge for healthcare systems, due to their propensity for adverse outcomes. This study has identified that, despite their recognised increased complications rates, mortality and cost implications, research into improving outcomes needs to be increased. A collaborative approach to overall bundle utilization in patients undergoing emergency laparotomy has identified an effective way of reducing mortality. Published consensus key performance indicators related to emergency laparotomy should be reported in future emergency laparotomy research, e.g., emergency surgery patients undergoing SSI surveillance; documentation of wound care bundle usage to include pre-operative, intra-operative, and post-operative key interventions; and having a documented laparotomy technique that includes facial, subcutaneous and skin to ensure reduction in adverse events [45].

To address the issues of SSI and adverse outcomes, wound bundles were recommended by the WHO and now are widely used in many areas of surgery [46]. Bundles with an increasing number of elements are shown to have the greatest effect [17].

Ariyo, in a recent systematic review of implementation strategies which aimed to reduce SSIs, found that out of 125 studies that met their inclusion criteria, only eight studies met the Effective Practice and Organization Care (EPOC) criteria. This limited their ability to identify the best interventions. In addition, many studies used multifaceted strategies to improve adherence with the evidence-based interventions, which posed a further challenge of interpretation [47].

Newly formed emergency surgical societies, such as The World Society of Emergency Surgery (WSES) and initiatives to improve outcomes, are addressing the lack of data in this area [4,48]. However, there remains the need for more robust clinical outcome data, registries and audits [4,49]. Variability in care also remains a huge challenge [50]. Many of the surgical colleges have advocated for new approaches to acute surgery, but uptake is slow [51,52]. Surgical site infection and outcomes from laparotomy, the focus of our particular systematic review, suffers from variable definitions of SSI from that of CDC to that of National Surgical Quality Improvement Project (NSQIP) [26]. The definition and heterogeneity of both superficial and deep SSIs constitutes a global challenge. DeBord, in an editorial review of the issue, looked at different ways to classify surgical site events and occurrences and suggested the creation of a joint task force to establish definitions for wound events. While the editorial referred to hernia repair, there is a ubiquitous need for this to also apply to EGS [53]. The incidence of SSI in colorectal surgery is generally between 15% to 30% [54,55]. It is clear from this current systematic review and meta-analysis that there is a need to standardise the use of clear definitions for future research on SSIs.

Given the paucity of studies relating to emergency laparotomy closure and bundles we felt abdominal domain closure, the “Surgeon’s signature”, is one of the keys to laparotomy. As surgeons, we understand that technical elements are important and Aicher’s recent multicentre study throws light on outcomes following emergency colorectal surgery; there was a 27.3% surgical site infection rate and 5.3% fascial dehiscence in 469 patients operated on in 21 medical centres in the US between 2018 and 2019 [56].

Regarding hyperoxygenation, it has been shown that there is a decrease in SSIs in patients receiving 80% FiO_2_ compared to 30% FiO_2_. However, this study had a very small sample group (n = 26) [31]. Hyperoxygenation has been a controversial treatment modality, with it potentially promoting pulmonary atelectasis. In 2016, the WHO highly recommended the use of high FiO_2_ in adult patients undergoing general anaesthesia in order to decrease the risk of SSI [57]. Since then, further research and discussion have highlighted issues with this recommendation, prompting the WHO to downgrade its recommendation in 2018 from strong to conditional. A recent meta-analysis from de Jonge et al. on the value of peri-operation hyperoxygenation found that high FiO_2_ (80%) was beneficial in intubated patients (RR = 0.80; 95% CI, 0.64–0.99)), but not in non-intubated patients (RR = 1.20; 95% CI, 0.91–1.58; p = 0.048) [58]. Thus, its selective use should be considered.

Incisional negative pressure wound therapy (iNPWT) is a relatively new therapy that has been used in many surgical fields, including general and colorectal surgery, for the prevention of SSIs. iNPWT is thought to promote angiogenesis, reduce oedema, increase tensile strength and reduce SSI [59]. The use of iNPWT, rather than primary suturing of the closure wound, showed a significant reduction in SSI [32]. iNWPT, when used in a bundle, may allow both clean contaminated and contaminated wounds to be closed primarily [60,61,62].

The effects of fluid level index were evaluated and it was shown that a lower tertile of fluid infusion index (FII) has a decreased risk of SSI and a higher tertile of FII was the most likely to cause SSI; however, both of these effects were shown to be insignificant [36]. The same study also examined the effects of temperature intra-operatively, highlighting that hypothermia (<35.4 °C) had a significant effect on SSI, whilst hyperthermia (>37.6 °C) had an insignificant effect on the rate of SSI [36]. The effectiveness of hypothermia compression showed a 12% decrease in SSI rates; however, that study was shown to be insignificant [37].

To determine the outcome of our study, we have pooled five grouped abdominal wall closure studies, which in themselves are limited by their small number and heterogenicity [33,34,35,38,40]. This grouping may not be justifiable and raises the increasingly obvious problem of lack of robust research in EGS surgical outcomes. A further limitation to our study was the availability of studies with small bundle numbers. These authors tested further new elements in conjunction with existing ill-defined bundle use, such as antibiotic administration. Previous authors have based recommendations on data extrapolated from elective settings [18].

We identify the grouping of the five studies into a technical closure bundle as a limitation; we did this to highlight the importance of the closure of the abdomen. As a key element of any bundle, we felt it was important to showcase its effect on SSI in EGS. Our analysis of bias in the included studies identified further limitations relating to the absence of relevant information on blinding, generation of allocation sequence, type of randomisation, allocation concealment, reasons for withdrawals and the numbers lost to follow-up. We also acknowledge the limitation of combining the number of overall, superficial and deep SSIs in our results. We did this to convey the numbers of SSIs in each category.

It is time for action. Robust, well designed and well defined multi-centre studies utilising wound bundles as part of clinical pathways, combined with safety programmes for improving surgical care and recovery, are required. Despite our research not showing significant results for wound bundles in EGS, it was hampered by the small numbers available. We believe, along with Eton and colleagues, that with further research, every EGS patient deserves pathway-aided care, which is inclusive of a wound bundle [63].

## Figures and Tables

**Table 1:** Characteristics of studies used in systematic review and meta-analysis.

Author and Year	Country	Study Design	Data Collection Period	Sample Size	SSI Definition	Type of SSI	Surveillance
Alvandipour 2019 [31]	Iran	Pro	Not Stated	26	ASEPSIS	Not stated	30 days
Danno 2018 [32]	Japan	Retro	19 months	47	CDC	Deep	Not Stated
Dayama 2018 [33]	USA	Retro	1 year	1792	NSQIP	Superficial and Deep	30 days
DeVries 2019 [34]	The Netherlands	Retro	3 years,	88	CDC	Superficial and Deep	Not Stated
Frazee 2017 [35]	USA	RCT	Not Stated	49	Not Stated	Deep	Not Stated
Gundersen 2018 [36]	Denmark	Retro	2 years	382	CDC	Not stated	14 days
Kiliç 2018 [37]	Turkey	RCT	1 year	100	CDC	Superficial	30 days
Peponis 2018 [38]	USA	RCT	7 years, 1 month	78	Not Stated	Not stated	Not Stated
Phelan 2019 [39]	England	Pro	Not Stated	83	CDC	Superficial and Deep	30 days
Ruiz-Tovar 2020 [40]	Spain	RCT	5 months	139	CDC	Deep	30 days
Yamamoto 2015 [41]	Japan	Retro	5 years	72	CDC	Deep	30 days

ASEPSIS = Additional treatment, the presence of Serous discharge, Erythema, Purulent exudate, and Separation of the deep tissues, the Isolation of bacteria, and the duration of inpatient Stay. NSQIp = National Surgical Quality Improvement Project. RCT = Randomised Control Trial.

**Table 2:** Experimental vs. control and outcome.

Author	Study Design (n = 2856)	Contributing Factor	Conclusion—SSI
Alvandipour (2019) [31]	Prospective N = 26	80% FiO_2_ O2 intra-op versus 30% FiO_2_ intra-op	RR = 1.93 (0.76–4.93) (p = 0.19)
Danno (2018) [32]	Retrospective N = 47	NPWT + Delayed primary closure versus primary closure	RR = 0.17 (0.06–0.52) (p = 0.002)
Dayama (2018) [33]	Retrospective N = 1792	Complete skin closure versus skin open	RR = 1.93 (0.76–4.93) (p = 0.17)
DeVries (2019) [34]	Retrospective N = 88	Small bite technique versus large bite technique	RR = 0.61 (0.36–1.04) (p = 0.07)
Frazee (2017) [35]	RCT N = 49	Incisional NPWT + open versus incisional NPWT + closed	RR = 0.52 (0.05–5.38) (p = 0.58)
Gundersen (2018) [36]	Retrospective N = 382	(1) Hyperthermia (>37.6) versus normothermia (35.5–37.5) (2) Hypothermia (<35.4) versus normothermia (35.5–37.5) (3) Fluid infusion index (FII) <2.7 versus FII 2.71–5.64 (4) FII >5.64 versus FII 2.71–5.64	(1) RR = 1.34 (0.73–2.45) (p = 0.34) (2) RR = 2.31 (1.30–4.08) (p = 0.004) (3) RR = 1.12 (0.62–2.02) (p = 0.72) (4) RR = 1.30 (0.79–2.15) (p = 0.3)
Kiliç (2018) [37]	RCT N = 100	Hypothermia compression versus normothermia compression	RR = 0.65 (0.34–1.24) (p = 0.19)
Peponis (2018) [38]	RCT N = 78	Interrupted fascial closure versus continuous fascial closure	RR = 1.33 (0.44–4.00) (p = 0.61)
Phelan (2019) [39]	Prospective N = 83	Bundle versus pre-bundle	RR = 0.94 (0.45–1.96) (p = 0.87)
Ruiz-Tovar (2020) [40]	RCT N = 139	Triclosan-coated barbed suture versus polydioxanone loop suture	RR = 0.39 (0.12–1.29) (p = 0.12)
Yamamoto (2015) [41]	Retrospective N = 72	Bundle versus pre-bundle	RR = 0.47 (0.20–1.10) (p = 0.12)
